# Duodenal Ulcer Perforation Following Tirzepatide Injections for Weight Loss Management: A Case Report

**DOI:** 10.7759/cureus.116411

**Published:** 2026-09-17

**Authors:** Hayder Al-Azzawi, Hugo Cohen

**Affiliations:** 1 Department of General Surgery, Great Western Hospital, Swindon, GBR; 2 Academic Department of Military Surgery and Trauma, Royal Centre for Defence Medicine, Birmingham, GBR

**Keywords:** adverse reactions, duodenal perforation, glp-1 receptor agonists, upper gastrointestinal surgery, weight reduction

## Abstract

We present a case of duodenal ulcer (DU) perforation occurring after dose escalation of tirzepatide for weight loss in a 43-year-old woman. She presented to the emergency department with colicky abdominal pain two weeks after increasing her tirzepatide dose from 2.5 mg to 5 mg once weekly. Computed tomography images demonstrated a localised retroperitoneal perforation of a DU involving the third portion of the duodenum. She was managed conservatively with intravenous antibiotics, proton pump inhibitor therapy and bowel rest. The patient improved clinically and was discharged.

While a causal relationship between tirzepatide and duodenal perforation cannot be established from this case alone, the close temporal relationship between dose escalation and presentation raises the question of an association between tirzepatide and upper gastrointestinal (GI) pathology. This case highlights the potential for this rare complication and supports the need for further evaluation of upper GI adverse events in patients receiving tirzepatide.

## Introduction

Tirzepatide, marketed under the brand name Mounjaro, is a dual agonist of glucose-dependent insulinotropic polypeptide (GIP) and glucagon-like peptide-1 (GLP-1) receptors [1]. It is licensed in the United Kingdom for the management of type 2 diabetes mellitus (T2DM). As of November 2023, tirzepatide has been licensed for the treatment of obesity in patients with a body mass index (BMI) ≥30 kg/m² [1,2]. Use of GLP-1 receptor agonists has increased exponentially in the UK, with one study predicting that 1 in 10 adults in the United Kingdom had either used a GLP-1 receptor agonist or were interested in using one in the future, with a large number of these prescriptions being obtained through the private sector [3].

Tirzepatide has three key mechanisms of action: enhancing insulin sensitivity and secretion, suppressing glucagon production, and delaying gastric emptying [1]. The delay in gastric emptying causes appetite suppression and early satiety, making it effective in weight management [2]. However, delayed gastric emptying may also contribute to adverse gastrointestinal (GI) effects [4].

The side effect profile of tirzepatide includes common GI symptoms such as nausea, vomiting and abdominal discomfort [1]. Less frequently, it has been associated with biliary complications and pancreatitis [1]. Although GI symptoms are well-recognised, reports of serious GI complications are rare. To date, only one case of duodenal ulcer (DU) perforation linked to tirzepatide use has been published [4].

The duodenum is the first part of the small intestine, connecting the pylorus of the stomach to the jejunum, the second part of the small intestine. Its walls are made of mucosa, submucosa, muscularis, and serosa [5,6]. The duodenum has important anatomical significance due to its proximity to the pancreas and nearby biliary and vascular structures [5]. The duodenum is susceptible to ulceration, which can, in some cases, lead to DU perforation, which is a hole in the wall of the duodenum [5,6]. The biggest risk factors for developing DU are *Helicobacter (H.) pylori *infection or non-steroidal anti-inflammatory drugs (NSAIDs). Other risk factors include smoking, stress, or corticosteroid use [2]. DU is not listed as a known side effect of tirzepatide on the British National Formulary (BNF) [1].

In the literature, there is one known case report that reports a DU perforation following tirzepatide treatment [4]. This case report presents similar findings.

## Case presentation

A 43-year-old woman presented to the emergency department (ED) with sudden-onset, severe epigastric and right upper quadrant pain (RUQ). Her medical history included obesity and anxiety, for which she was taking fluoxetine. She was otherwise fit and well. She measured at 162 cm tall and was reported to weigh 95 kg before starting the medication. Her BMI would have been 36.2 kg/m2. She had been self-administering over-the-counter tirzepatide (Mounjaro) injections for weight loss over the previous six weeks. She was adherent to a once-weekly regimen, initially taking 2.5 mg for 4 weeks, after which she increased the dose to 5 mg weekly. She reported having lost 9 kilograms during this period.

Approximately two weeks after the dose escalation, she developed abrupt epigastric and RUQ pain at 22:00 hours, prompting presentation to the ED at 09:00 the following morning. She described the pain as colicky with radiation to the back. It reached maximum intensity immediately upon onset and was exacerbated by movement and palpation. She reported nausea but no vomiting.

Initial observations in ED were as follows: temperature 36.8 °C, respiratory rate 17 breaths/min, oxygen saturation 99% on room air, heart rate 89 bpm, and blood pressure 122/78 mmHg. Her inflammatory markers and blood tests showed leucocytosis and neutrophilia: white blood cells 19 × 10⁹/L, neutrophils 17 × 10⁹/L. Her C-reactive protein was 4 mg/L, serum lactate 1.1 mmol/L. Her liver function, amylase, and renal function were all within normal limits.

The patient was in significant pain, requiring multiple doses of IV opioids to facilitate clinical examination. Her abdomen was soft, with localised tenderness and guarding in the epigastric region.

Due to the severity of the pain, an urgent contrast-enhanced CT scan of the abdomen and pelvis was performed. CT showed a localised retroperitoneal DU perforation involving the third portion of the DU (D3). See Figures 1, 2.

**Figure 1:**
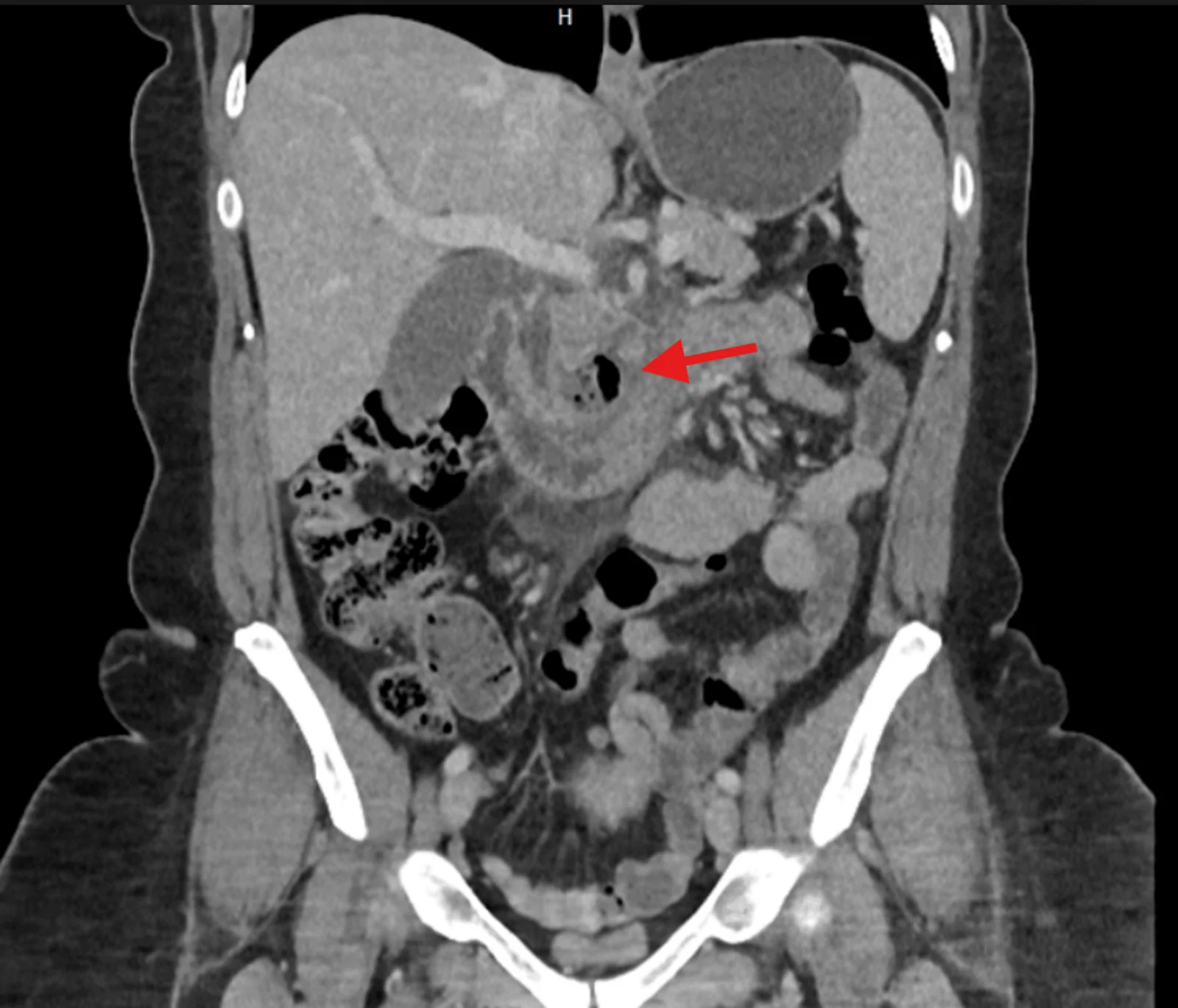
Coronal CT image showing a gas-filled cavity (red arrow) adjacent to the third part of the duodenum (D3) within the lesser sac, consistent with a perforation

**Figure 2:**
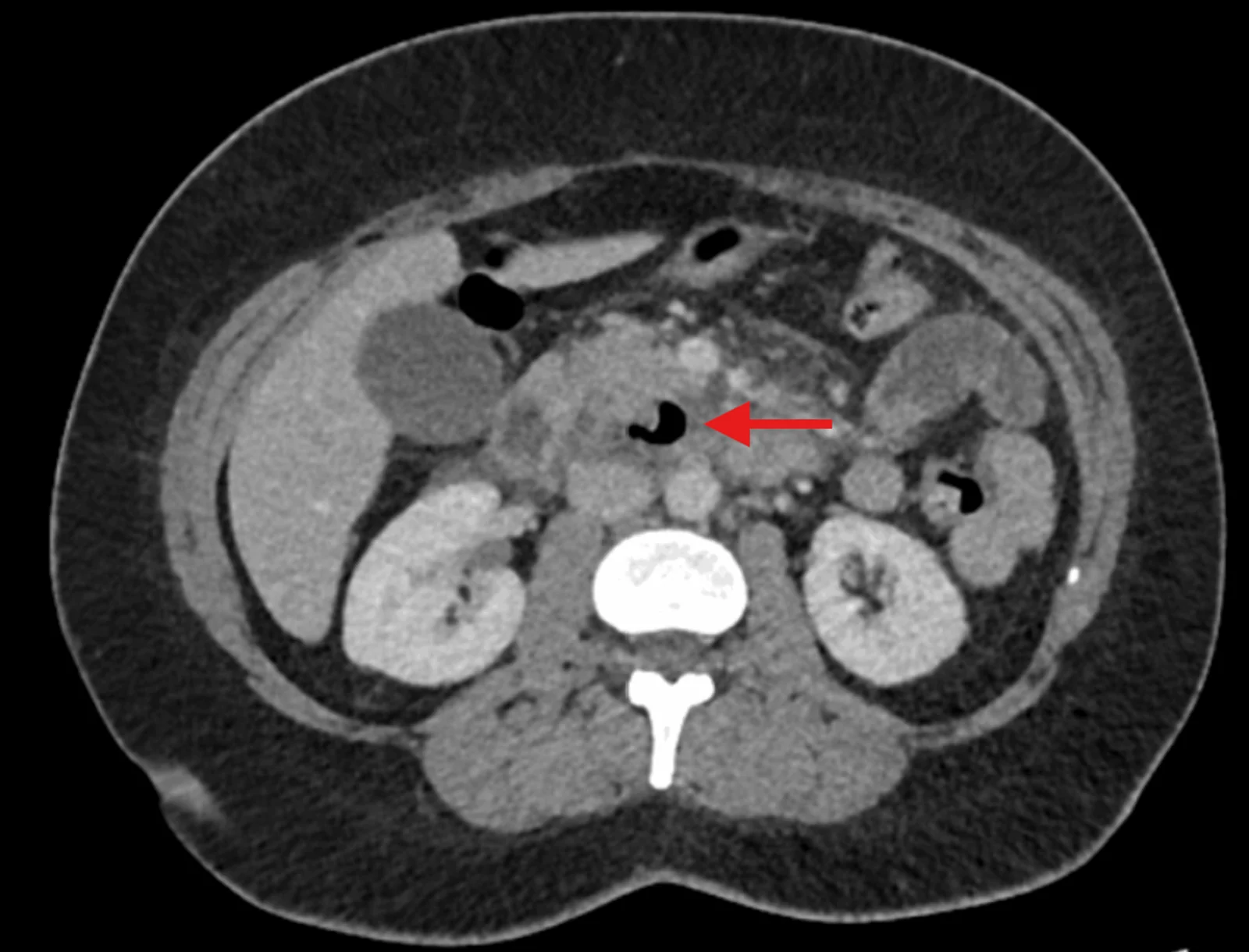
Sagittal CT image showing a gas-filled cavity (red arrow) adjacent to the third part of the duodenum (D3) within the lesser sac, consistent with a perforation

Due to the retroperitoneal location of the perforation and the absence of generalised peritonitis, the patient was managed conservatively. She received intravenous fluids, high-dose proton pump inhibitor therapy (omeprazole 40 mg IV, twice a day), and broad-spectrum intravenous antibiotics. She had a nasogastric tube inserted and was kept nil by mouth for three days. Oral intake was gradually reintroduced following this period of observation and clinical improvement.

*H. pylori* status was not assessed at admission or at subsequent follow-up. This represents a major limitation of this case report. This was largely due to clinical oversight and due to the patient being started on high-dose proton pump inhibitors and antibiotics, which would reduce the sensitivity of *H. pylori *results.

The patient made a swift recovery and was discharged in stable condition by day 5 with instructions to continue oral PPI therapy. An outpatient oesophagogastroduodenoscopy (OGD) was performed two months later; the OGD had good views of the duodenum and showed no evidence of active duodenal pathology. Views of D1-D4 are seen in Figure 3. The patient was discharged from our care with advice on the use of PPI while continuing the use of tirzepatide injections. *H. pylori *status was not measured during this admission or at a biopsy taken at the OGD.

**Figure 3:**
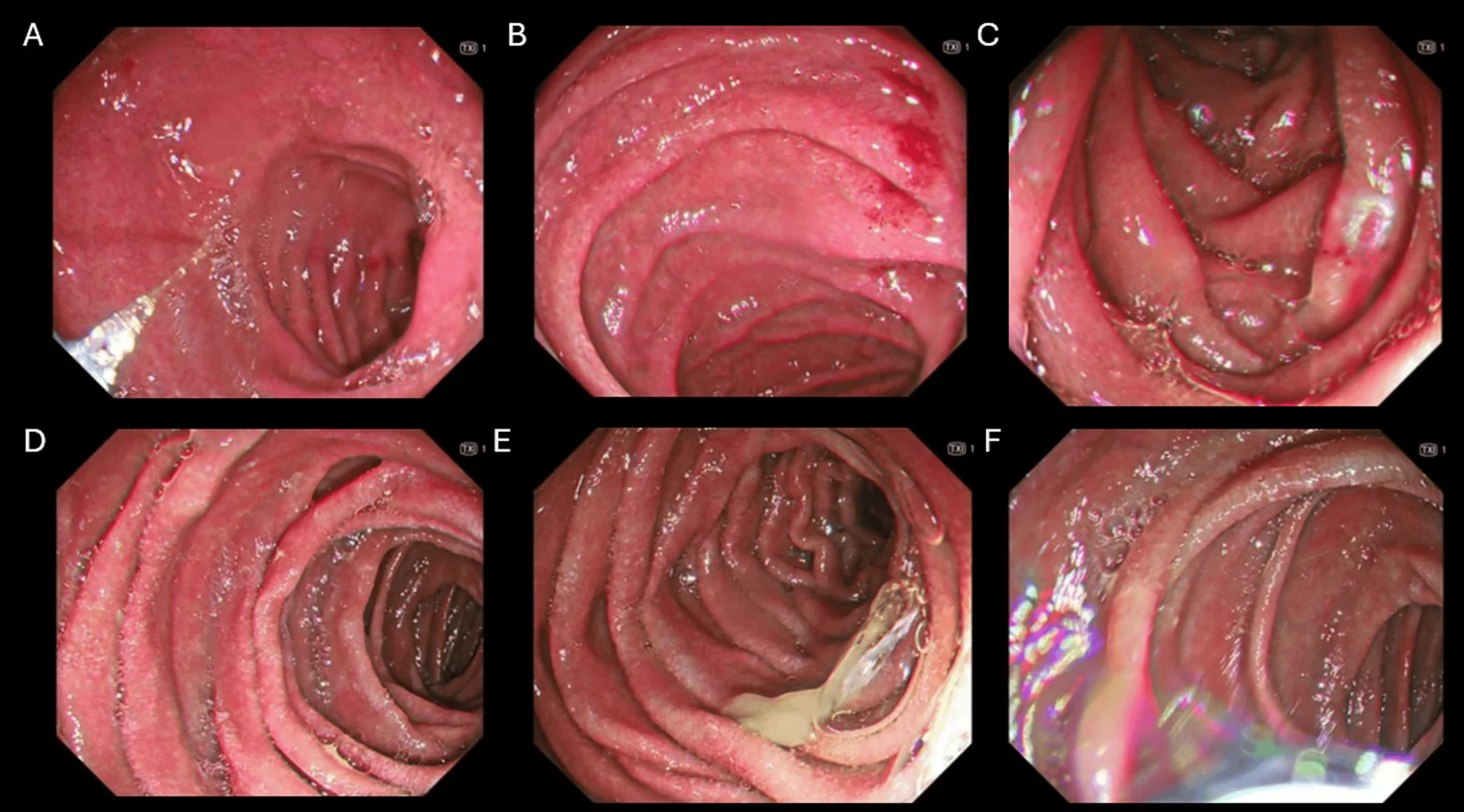
OGD images A. Duodenal bulb, B. Duodenum: first part, C. Duodenum: second part, D. Duodenum: third part 1, E. Duodenum: third part 2, F. Duodenum: fourth part All images show normal mucosa and no signs of duodenal perforation at two months. OGD: oesophagogastroduodenoscopy

## Discussion

We report a case of DU perforation associated with the use of tirzepatide. While the *H. pylori* status of the patient was not known, the patient had no known risk factors for DU perforation and no prior symptoms suggestive of peptic ulcer disease (PUD) before the acute presentation.

A limitation of this case report is the lack of data regarding the patient’s *H. pylori* status. *H. pylori *is a known risk factor for the development of PUD [5-7]. The patient was started on a PPI and antibiotics that would eradicate *H. pylori*. Both of these would affect the sensitivity of *H. pylori *tests. Unfortunately, due to a clinical oversight, this test was not completed during our patient’s admission or at follow-up OGD. An explanation for this patient’s symptoms and presentation could entirely be linked to the undetected *H. pylori *infection.

While the absence of *H. pylori *testing presents a major limitation in the conclusions that can be drawn from this case, the close temporal relationship of the timeframe between taking tirzepatide and the onset of symptoms should still be acknowledged. 

Given the lack of pre-existing GI symptoms and the association with the initiation and dose escalation of tirzepatide, we propose that tirzepatide may have contributed to the development of DU perforation in this case. GI side effects, including nausea, vomiting, and delayed gastric emptying, are well-documented adverse effects of tirzepatide [1]. However, there is only one previously reported case in the literature that links tirzepatide to DU perforation [4].

While the temporal relationship alone does not establish a drug-induced injury, we propose the following theoretical mechanisms that support a link between GLP-1 receptor agonists and peptic ulcer complications. GLP-1 is known to delay gastric emptying, which may increase duodenal mucosal exposure to gastric acid [8]. Tirzepatide contains both GLP-1 and GIP receptor agonists [1]. The GIP component also causes delayed gastric emptying and reduced gastric motility. Conversely, GIP can have a gastric protective element, as it inhibits gastric acid secretion through the suppression of gastrin release [9]. The rationale for combining GLP-1 and GIP agonism lies in their potential to balance GI side effects by reducing acid secretion to balance the reduction in gastric motility [10].

Although a definitive causal relationship between tirzepatide and DU perforation cannot be established from a single case, the timing of symptom onset following initiation and dose escalation, combined with similar findings, raises concern. These observations warrant further investigation. Future studies are needed to assess whether there is a clinically significant association between tirzepatide use and the development of peptic ulcer disease and its complications.

## Conclusions

This case highlights the potential association between tirzepatide and DU perforation, especially within the limitations of having only one patient and lacking *H. pylori* data. While there are protective factors in the use of the GIP/GLP-1 combination by reducing gastric secretion as well as gastric motility, the combined impact on delayed gastric emptying and reduced GI motility may predispose patients to peptic ulcer complications. While a causal link cannot be confirmed based on a single case, this report represents the second reported instance of DU perforation associated with tirzepatide therapy. Given the known side effect profile of tirzepatide causing GI disorders and the temporal relationship between dose increase and onset of symptoms, we recommend that clinicians be aware of the possible risk factors when assessing patients and emphasise that further evidence is required to identify if there is an increased risk of DU associated with tirzepatide use.
